# Loss of CD28 expression associates with severe T-cell exhaustion in acute myeloid leukemia

**DOI:** 10.3389/fimmu.2023.1139517

**Published:** 2023-03-07

**Authors:** Yueting Huang, Huijian Zheng, Yuwen Zhu, Yan Hong, Jie Zha, Zhijuan Lin, Zhifeng Li, Caiyan Wang, Zhihong Fang, Xingxing Yu, Long Liu, Bing Xu

**Affiliations:** ^1^Department of Hematology, The First Affiliated Hospital of Xiamen University and Institute of Hematology, School of Medicine, Xiamen University, Xiamen, China; ^2^Key Laboratory of Xiamen for Diagnosis and Treatment of Hematological Malignancy, Xiamen, China

**Keywords:** CD28, acute myeloid leukemia, exhaustion, T cell, senescence

## Abstract

**Introduction:**

Despite accumulated evidence in T-cell exhaustion in acute myeloid leukemia (AML), the immunotherapeutic targeting exhausted T cells such as programmed cell death protein 1 (PD-1) blockade in AML failed to achieve satisfying efficacy. Characteristics of exhausted T cells in AML remained to be explored.

**Methods:**

Phenotypic analysis of T cells in bone marrow (BM) using flow cytometry combining senescent and exhausted markers was performed in de novo AML patients and healthy donors as well as AML patients with complete remission (CR). Functional analysis of T-cell subsets was also performed in de novo AML patients using flow cytometry.

**Results:**

T cells experienced a phenotypic shift to terminal differentiation characterized by increased loss of CD28 expression and decrease of naïve T cells. Additionally, lack of CD28 expression could help define a severely exhausted subset from generally exhausted T cells (PD-1^+^TIGIT^+^). Moreover, CD28- subsets rather than CD28+ subsets predominantly contributed to the significant accumulation of PD-1^+^TIGIT^+^ T cells in AML patients. Further comparison of de novo and CR AML patients showed that T-cell exhaustion status was improved after disease remission, especially in CD28+ subsets. Notably, higher frequency of CD28-TIGIT-CD4^+^ T cells correlated with the presence of minimal residual disease in AML-CR group. However, the correlation between CD28- exhausted T cells and cytogenetic risk or white blood cell count was not observed, except for that CD28- exhausted CD4^+^ T cells correlated with lymphocyte counts. Intriguingly, larger amount of CD28-TGITI^+^CD8^+^ T cells at diagnosis was associated with poor treatment response and shorter leukemia free survival.

**Discussion:**

In summary, lack of CD28 expression defined a severely exhausted status from exhausted T cells. Accumulation of CD28- exhausted T cells was linked to occurrence of AML, and correlated to poor clinical outcome. Our data might facilitate the development of combinatory strategies to improve the efficacy of PD-1 blockade in AML.

## Introduction

Immunotherapies revolute the therapeutic models of cancer by improving anti-tumor immunity, especially T cell function. However, the outcome of clinical trials using immunotherapies targeting T cells such as programmed cell death protein 1 (PD-1) blockade in acute myeloid leukemia (AML) remain frustrating (1). Hence, a more detailed analysis of T cell status, especially T-cell exhaustion in AML is warranted to help us envision more tailored immunotherapeutic strategies for the future of AML treatment.

Due to persistent antigen stimulation in tumors or chronic infections, T cells acquire an exhausted phenotype accompanied by a loss of function (2). T cell exhaustion is characterized by impaired function and high expression of immune checkpoint inhibitory receptors such as PD-1, T-cell immunoglobulin and immunoreceptor tyrosine–based inhibitory motif (ITIM) domain (TIGIT) (3, 4). As we and others reported, exhausted T cells were accumulated in AML patients (4–8). However, PD-1 blockade treatment targeting exhausted T cells in AML showed poor efficacy (1). Severity of T-cell exhaustion correlate to the response to immune check point inhibitors (ICIs) (9). Severe exhaustion of T cells is responsible for the failure of ICIs treatment (9). Coexpression of multiple inhibitory receptors such as PD-1, T cell immunoglobulin and mucin domain 3 (TIM-3), Lymphocyte activation gene-3 (LAG-3) and TIGIT or high expression of PD-1 were used to define severely exhausted T cells in the previous studies (9, 10). Nevertheless, inhibitory receptors are upregulated in activated T cells besides exhausted T cells (11). Moreover, different combinations of inhibitory receptors might incur an inconsistent result. Additionally, due to the consecutive expression of PD-1 on T cells, gating PD-1^hi^ subset of T cells is rather subjective. More markers should be involved to distinguish the exhaustion status in AML.

In tumor settings, T cells could experience a phenotypic shift to terminal differentiation characterized by expressing KLRG-1 and CD57 but loss of CD28 after persistent exposure to neoantigen, which is termed “T-cell senescence” (12). Moreover, proliferative arrest and shortened telomeres are also the characteristic features of senescent T cells. Although senescence and exhaustion mechanically undergo different molecular process, these two states of T cells overlap with regard to the expression of several phenotypic markers (6, 12). As shown in multiple myeloma, T cells with senescent markers like CD57 also express PD-1 (13). As reported, exhaustion of T cells could be largely reversed by ICIs while senescence of T cells is considered as an irreversible process (14). Intriguingly, Kamphorst AO et al. found that CD28 negative exhausted T cells cannot be rescued by PD-1 blockade therapy (15). Recently, George Coukos et al. also demonstrated that tumor-infiltrated T cells activation on anti-PD-1 therapy depends on CD28 costimulation catered by myeloid APCs *in situ* of ovarian cancer (16). These data indicated that CD28 expression might be critical for active T cell response in tumor. Additionally, the IL-7/IL-7Rα axis is involved in the maintenance of naïve and long-living memory cells as well as self-renewal in T cell populations (5, 17, 18). Defect of IL-7 signaling is not only involved in exhaustion but also plays a crucial role in senescence of T cells (17, 19).

In this study, we provide a comprehensive analysis of the expression of senescence-related markers such as CD28, KLRG-1 and CD57 and define severely exhausted T cells using CD28 combining with exhausted markers like PD-1 and TIGIT in bone marrow (BM) samples from AML patients. Since AML blasts as a key role in the compromising immune response are heterogenous and vary along with disease status, the alterations of severely exhausted T cells in different stages of the disease and the correlation to clinical features and prognosis were also evaluated.

## Material and methods

### Patients and clinical evaluation

Bone marrow (BM) of 42 *de novo* AML patients and 32 AML patients in complete remission (CR) as well as from healthy donors (HDs, n = 15) were collected after written informed consent was signed in accordance with the Declaration of Helsinki and approved by the ethics board of the First Affiliated Hospital of Xiamen University. All samples were taken from non-acute promyelocytic leukemia (APL) AML patients. Positivity of minimal residual disease (MRD) was defined as AML cells detected by real-time quantitative polymerase chain reaction (RQ-PCR) or multicolor flow cytometry in BM samples. CR, CR with in complete hematologic recovery (CRi) and partial remission (PR) and no response (NR, induction failure) were defined according to 2017 ELN recommendations (20). Risk stratification by genetics in AML was performed according to 2017 ELN recommendations (20).

### Multiparameter flow cytometry analysis

Surface staining was immediately performed after BM collection. A 100μl BM sample was incubated with directly conjugated monoclonal antibodies for 15 minutes at room temperature; after lysing red blood with 1ml lysing solution (BD Bioscience), the cells were washed and resuspended by phosphate buffered saline (PBS). The monoclonal antibodies used were anti-human CD3-Percp (UCHT1), CD28-FITC (CD28.2), CD4-APC-R700 (RPA-T4), CD8-APC-R700 (RPA-T8), CD45RA-APC-CY7 (HI100), CCR7-BV421 (G043H7), PD-1-PE-Cy7 (EH12.1), CD127-BV510 (A019D5), CD57-PE (HCD57), KLRG-1-APC (SA231A2), TIGIT-BV605 (A15153G) from Biolegend, CA, USA.

### Isolation of bone marrow mononuclear cells and ex-vivo stimulated cytokines production and cytotoxic potentials

BM was diluted 1:1 with PBS before separation of bone marrow mononuclear cells (BMMCs) with lymphocyte separation solution (TBD Science, China) density gradient centrifugation. The isolated BMMCs were cultured in RPMI-1640 medium (GIBCO) containing 10% fetal bovine serum and stimulated with anti-CD3/CD28 antibody (2 and 5μg/ml) or Phorbol-12-myristate-13-acetate (PMA, 100ng/ml) and ionmycin (1ug/ml) for 5 h. Anti-CD107a-APC (W18263B), CD28-FITC and Golgiplug (BD Pharmingen, San Diego, CA, USA) were added at the start of the incubation. Cells were harvested for surface staining with CD3-Percp, CD8-APCR700, CD45RA-APC-H7, CCR7-V450, PD-1-PE-Cy7, TIGIT-BV605 and intracellularly stained with IFN-γ-BV510(4S.B3).

### Statistical analysis

All flow cytometric data were analyzed using Flowjo 10. software. All data were subjected to normal distribution analysis by the Kolmogorov-Smirnov test using SPSS 22.0 (SPSS, Inc., Chicago, IL, USA). Student’s t test was used to analyze data with a normal distribution, while the Mann-Whitney U test or Wilcoxon matched-pairs signed rank test (in paired setting) was used to analyze data without a normal distribution. Fisher’s exact test or chi-square test were used to analyze the constituent ratios between *de novo* AML and AML-CR group. P-values <0.05 were considered significant, where *, ** and *** indicated p-values between 0.01 to 0.05, 0.001 to 0.01 and 0.0001 to 0.001, respectively.

## Results

### Patient characteristics

Fifteen HDs (with a median age of 34.5 years, range 25–56 years), forty-two *de novo* AML patients and 32 AML patients in CR (AML-CR) were enrolled in the present study. The characteristics of *de novo* AML patients and AML-CR were summarized in **Table 1**. Five patients rejected further medical care and three patients died before the evaluation. Two patients dropped out due to transfer to other hospitals. Compared to *de novo* AML group, the percentages of patients at low risk and received idarubicin and cytarabine (IA) regimens tended to be higher in AML-CR group. Fewer patients in *de novo* AML group received low intensity chemotherapy in comparison to AML-CR group. Among 32 *de novo* AML patients, 18 patients achieved CR or CRi while 13 patients showed no response to induction therapy.

**Table 1:** Patient characteristics.

Characteristics	De novo AML (n=42)	AML-CR (n=32)	P value
Gender			1.000
Male (%)	24 (57.1)	19 (59.3)	
Female (%)	18 (42.9)	13 (40.7)	
Age ,years, median (range)	53 (14-83)	49 (14-78)	0.869
WBC(×10^9^/L) at diagnosis	8.32(0.43-189.48)	6.32(0.43-179.34)	0.782
Cytogenetic risk (%)			
Low	9 (21.4)	11(33.3)	
Intermediate,	15(35.7)	13 (40.6)	
High	13(31.0)	8 (26.1)	0. 278
NA	5 (11.9)	0	
Induction regimens			
HMA+Venetoclax based regimen	6(14.3)	4(12.5)	
Low dose chemotherapy	5(11.9)	8(25)	
IA based regimen	24(57.1)	20(62.5)	0.937
NA	7(16.7)	/	
Response after induction			
CR+CRi	18 (42.9)	/	
PR	1(2.4)	/	
NR	13(30.9)	/	
NA	10(23.8)	/	
Chemotherapy Cycles before BM collection	/	3 (2-5)	

WBC indicate white blood cell count; NA, not available; HMA, hypomethylation agents; IA, idarubicin + cytarabine; BM, bone marrow; AML, acute myeloid leukemia; AML-CR, patients with acute myeloid leukemia in compete remission; CR, complete remission; Cri, CR with incomplete hematologic recovery; PR, partial remission; NR, no response.

### Loss of CD28 expression is linked to terminal differentiation and senescence of T cells in *de novo* AML

**Figure 1:**
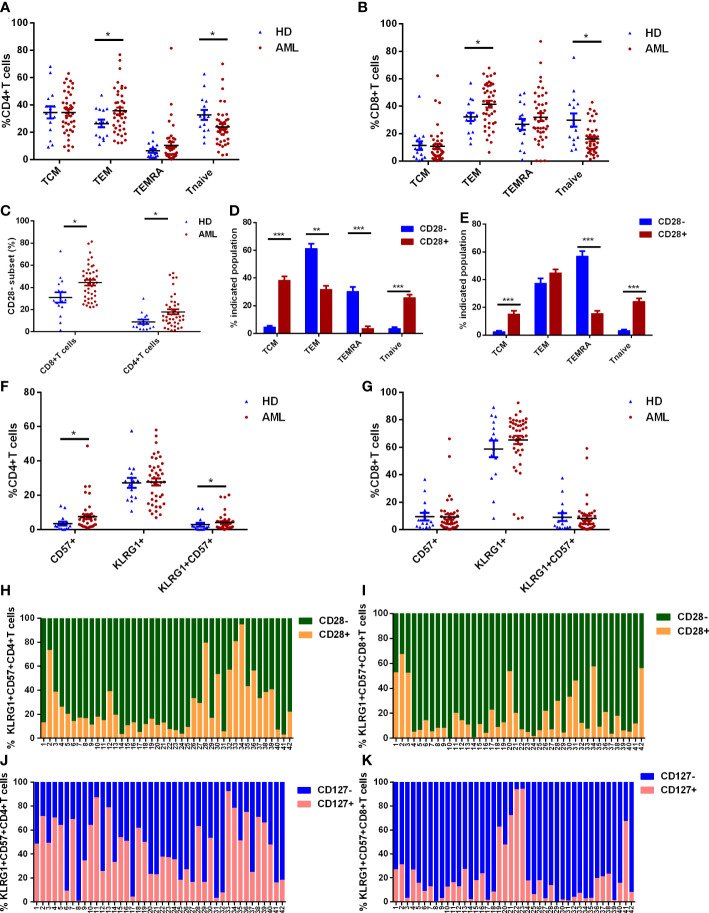
T cell differentiation and expression of senescent markers on T cells in *de novo* AML patients (n=42) and healthy donors (HDs, n=15). **(A, B)** The frequencies of naïve T cells (Tnaive, CCR7^+^CD45RA^+^), central memory T cells (TCM, CCR7^+^CD45RA^-^), effector memory T cells (TEM, CCR7^-^CD45RA^-^) and effector memory T cells with expression of CD45RA (TEMRA, CCR7^-^CD45RA^+^) defined by CCR7 and CD45RA in CD4^+^ **(A)** and CD8^+^ **(B)** T cells from bone marrow (BM) were compared in *de novo* AML and HDs. Additionally, the percentages of CD28^-^ subset in CD4^+^ and CD8^+^ T cells from BM of *de novo* AML and HDs were evaluated **(C)**. The differentiated status was further analyzed in CD28^-^ and CD28^+^ subset of CD4^+^ **(D)** and CD8^+^ T cells **(E)**. Senescent markers: CD57 and KLRG-1 expression on CD4^+^ **(F)** and CD8^+^ T cells **(G)** in BM were compared between AML patients and HDs. At last, CD28 expression in KLRG-1^+^CD57^+^ subset in CD4^+^ T cells **(H)** and CD8^+^ T cells **(I)** and CD127 expression in KLRG-1^+^CD57^+^ subset in CD4^+^ T cells **(J)** and CD8^+^ T cells **(K)** were analyzed in *de novo* AML patients. Student’s t test or Mann-Whitney U test was used to analyze the data according to normal distribution of data. *, ** and *** indicated p-values between 0.01 to 0.05, 0.001 to 0.01 and 0.0001 to 0.001, respectively.

T cell differentiation was evaluated in BM samples from *de novo* AML patients (n=42) and HDs (n=15). A significant phenotypic shift to effector memory T cells (TEM) was observed in *de novo* AML patients (**Figures 1A, B**). Moreover, both CD8^+^ and CD4^+^ naïve T cells (Tnaïve) from *de novo* AML patients suffered a significant shrinkage compared with HDs (**Figures 1A, B**). The frequencies of central memory T cells (TCM) and terminal effector memory expressing CD45RA^+^ (TEMRA) in CD4^+^ and CD8^+^ T cells were comparable in two groups (**Figures 1A, B**). Besides, more T cells lost CD28 expression in AML group compared with HDs (**Figure 1C**). A comparison of CD28^+^ and CD28^-^ T cells revealed that loss of CD28 was linked to late stage (TEM in CD4^+^ T cells) or even terminal differentiated stage (TEMRA in CD4^+^ and CD8^+^T cells) of T cells rather than early stage such as Tnaive and TCM (**Figures 1D, E**). Late or terminal differentiation were generally associated with T-cell senescence. Further analysis showed that significantly increased CD57^+^CD4^+^ T cells and KLRG-1^+^CD57^+^CD4^+^ T cells were observed in AML group (**Figure 1F**). However, no difference was observed in CD8^+^ T cell compartments (**Figure 1G**). Lack of CD28 expression was also observed in KLRG-1^+^CD57^+^ subsets in both CD4^+^ and CD8^+^ T cells (**Figures 1H–K**).

### Differentiation status defined by CCR7 and CD45RA could not distinguish a terminal exhausted T-cell subset associated with AML occurrence

**Figure 2:**
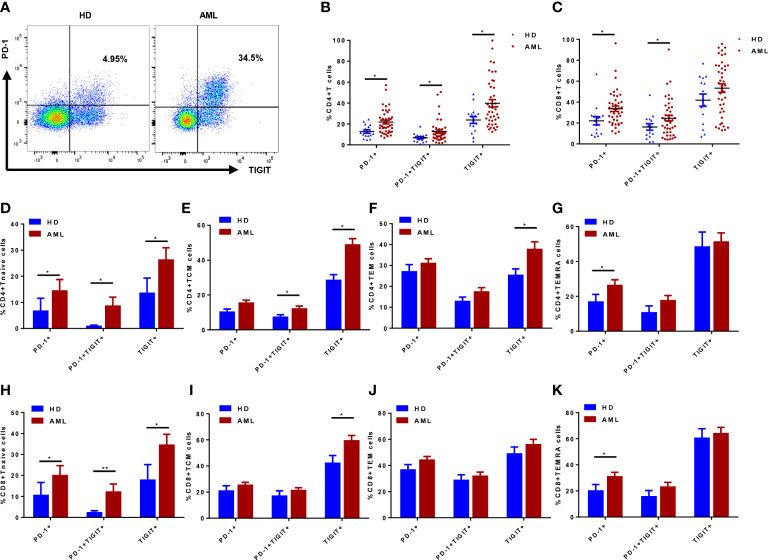
Inhibitory receptors PD-1 and TIGIT expression in T cells and their differentiated subsets from bone marrow (BM) of *de novo* AML patients (n=42) and healthy donors (HDs, n=15). PD-1 and TIGIT expression was determined in *de novo* AML patients and HDs **(A)**; The percentages of PD-1^+^, TIGIT^+^ and PD-1^+^TIGIT^+^ subsets of CD4^+^ **(B)** and CD8^+^ **(C)** T cells in BM of *de novo* AML patients and HDs were compared. Moreover, PD-1, TIGIT and coexpression of PD-1 and TIGIT were evaluated in Tnaive, TCM, TEM and TEMRA subsets of CD4^+^ **(D–G)** and CD8^+^ **(H–K)** T cells in BM of *de novo* AML patients and HDs. Student’s t test or Mann-Whitney U test was used to analyze the data according to normal distribution of data. *, ** indicated p-values between 0.01 to 0.05, 0.001 to 0.01 respectively.

The expression pattern of inhibitory receptors (including PD-1 and TIGIT) was also evaluated in AML patients and HDs (**Figure 2A**). In comparison to HDs, significantly higher percentages of PD-1^+^ and PD-1^+^TIGIT^+^ subsets were observed in both CD4^+^ and CD8^+^ T cell compartment of AML patients (**Figures 2B, C**). Although the percentage of TIGIT^+^ subset in both CD4^+^ and CD8^+^ T cells increased in AML patients, only the subset in CD4^+^ T cells presented a significant difference (**Figures 2B, C**). However, when we further analyzed the PD-1 and TIGIT expression pattern in distinctly differentiated subsets like Tnaive, TCM, TEM and TEMRA, the difference varied along with the differentiated status. Higher expression of PD-1 was observed in Tnaive and TEMRA subsets in both CD4^+^ (**Figures 2D, G**) and CD8^+^ T cell compartment (**Figures 2H, K**). Upregulated TIGIT expression was distributed in relatively early stage in Tnaive and TCM of CD4^+^ (**Figures 2D, E**) and CD8^+^ T cells (**Figures 2H, I**) as well as in CD4^+^ TEM (**Figure 2F**). However, a significant increase of T cells coexpressing of PD-1 and TIGIT was observed in CD4^+^ and CD8^+^ Tnaive (**Figures 2D, H**) and CD4^+^ TCM (**Figure 2E**). Neither TEMRA nor TEM showed a significant difference in coexpression of PD-1 and TIGIT in CD4^+^ (**Figures 2F, G**) or CD8^+^ T cells (**Figures 2J, K**). Taken together, these data suggested it might be hard to further classify exhausted T cells by combination of differentiated markers CCR7, CD45RA and inhibitory receptors PD-1 and TIGIT in BM of *de novo* AML patients.

### CD28 expression could phenotypically classify exhausted T cells in the BM of *de novo* AML patients

**Figure 3:**
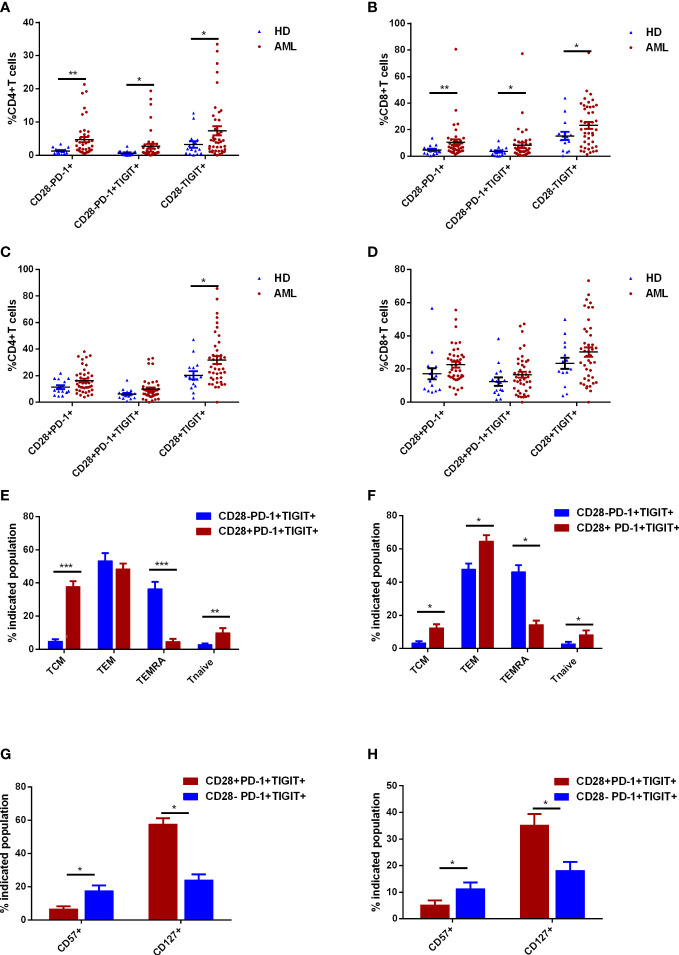
Further classification of exhausted T cells in the bone marrow (BM) of *de novo* AML patients using CD28 expression. The frequencies of CD28^-^PD-1^+^, CD28^-^PD-1^+^TIGIT^+^, and CD28^-^TIGIT^+^ subsets in CD4^+^ T cells **(A)** and CD8^+^ T cells **(B)** in BM were analyzed in *de novo* AML patients (n=42) and healthy donors (HDs, n=15). The frequencies of CD28^+^PD-1^+^, CD28^+^PD-1^+^TIGIT^+^, and CD28^+^TIGIT^+^ subsets in CD4^+^ T cells **(C)** and CD8^+^ T cells **(D)** in BM were further evaluated. Thereafter, the differentiated status of CD28^-^PD-1^+^TIGIT^+^ and CD28^+^PD-1^+^TIGIT^+^ subsets of CD4^+^ T cells **(E)** and CD8^+^ T cells **(F)** in BM were determined in AML patients. Moreover, CD127 and CD57 expression were also evaluated in CD28^-^PD-1^+^TIGIT^+^ and its counterpart in CD4^+^ T cells **(G)** and CD8^+^ T cells **(H)** in BM of *de novo* AML patients. Student’s t test or Mann-Whitney U test was used to analyze the data according to normal distribution of data. *, ** and *** indicated p-values between 0.01 to 0.05, 0.001 to 0.01 and 0.0001 to 0.001, respectively.

CD28^-^ exhausted T cells were defined as the functionally irreversible subpopulation by ICIs in chronic infection settings. The role of CD28 in classification of exhausted T cells in *de novo* AML patients remained to be assessed. In CD4^+^ T cell compartment, significant augment of PD-1^+^ and PD-1^+^TIGIT^+^ subsets were observed in CD28^-^ rather than CD28^+^ subset of AML patients compared with HDs (**Figures 3A, C**). However, TIGIT expression was consistently upregulated in CD28^-^ and CD28^+^CD4^+^ T cells (**Figures 3A, C**). As for CD8^+^ T cells, significantly higher percentages of PD-1^+^, TIGIT^+^ and PD-1^+^TIGIT^+^ in AML patients were merely observed in CD28^-^ T cells (**Figures 3B, D**). Additionally, CD28^-^PD-1^+^TIGIT^+^ T cells also displayed a late and terminal differentiated status compared with its CD28^+^ counterpart (**Figures 3E, F**). Moreover, other senescent markers like CD57 robustly increased in CD28^-^PD-1^+^TIGIT^+^ T cells compared with CD28^+^PD-1^+^TIGIT^+^ T cells (**Figures 3G, H**). Similarly, lack of CD127 expression was more frequently observed in CD28^-^PD-1^+^TIGIT^+^ T cells (**Figures 3G, H**). In summary, CD28 expression could phenotypically classify an exhausted T-cell subset with senescent features that correlated to AML occurrence.

### Lack of CD28 expression defines a severely functionally exhausted T cells in the BM of *de novo* AML patients

**Figure 4:**
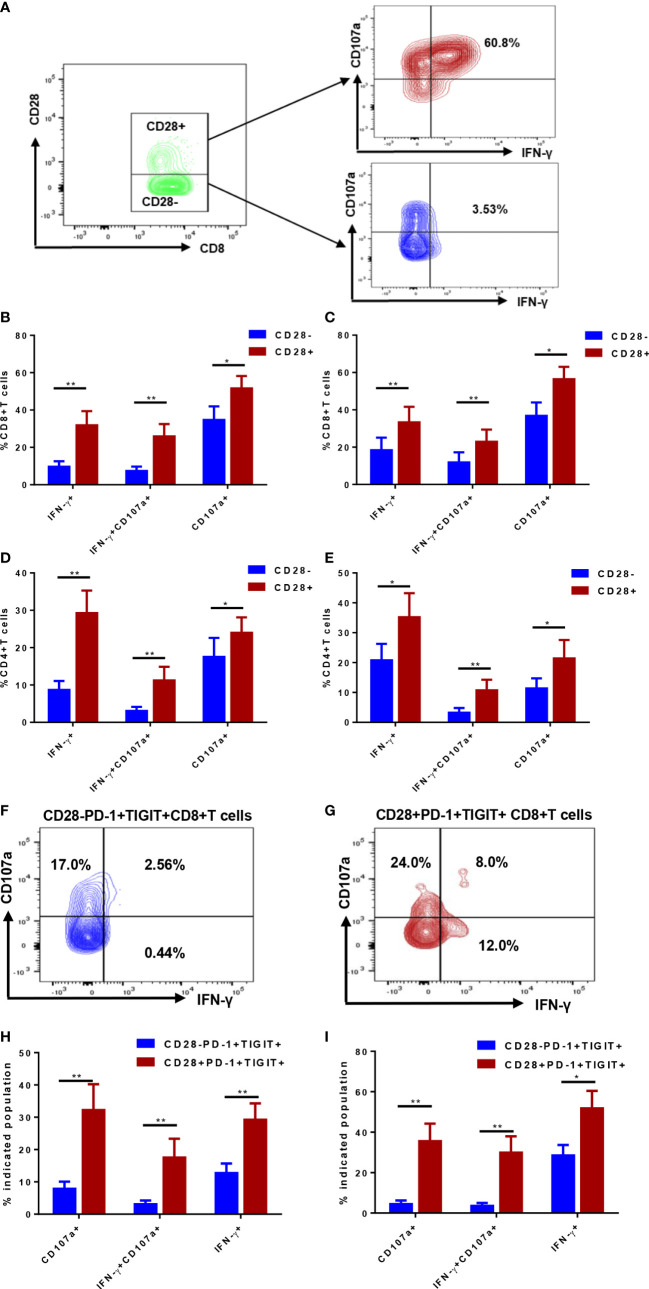
Function analysis of CD28^-^ subsets and the counterparts of T cells in in the bone marrow (BM) of *de novo* AML patients. Degranulation capacity and interferon-gamma (IFN-γ) were determined in CD28^-^ and CD28^+^ T-cell subsets **(A)**; CD107a expression and IFN-γ production were simultaneously evaluated in CD28^-^ and CD28^+^ subsets of CD4^+^ T cells **(B)** and CD8^+^ T cells **(C)** stimulated by anti-CD3/CD28 antibody ex vivo (n=17). Additionally, CD107a expression and IFN-γ production were simultaneously evaluated in CD28^-^ and CD28^+^ subsets of CD4^+^ T cells **(D)** and CD8^+^ T cells **(E)** stimulated by PMA/ionomycin ex vivo (n=13). Further analyses were performed to evaluate CD28^-^PD-1^+^TIGIT^+^CD8^+^ **(F)** and its counterpart **(G)** of CD8^+^ T cells; CD107a expression and IFN-γ production were evaluated in CD28^-^PD-1^+^TIGIT^+^ and its counterpart of CD4^+^ T cells **(H)** and CD8^+^ T cells **(I)** stimulated by anti-CD3/CD28 antibody ex vivo (n=17). Paired Student’s t test or Wilcoxon matched-pairs signed rank test was used to analyze the data according to normal distribution of data. *, ** indicated p-values between 0.01 to 0.05, 0.001 to 0.01 respectively.

Despite obvious distinction in phenotype in CD28^-^ and CD28^+^ T cells, whether lack of CD28 contributes to impairment in function remains to be elucidated. The capacity of degranulation and interferon-gamma (IFN-γ) production were evaluated in CD28^-^ and CD28^+^ T cells (**Figure 4A**). In an ex vivo simulation setting using CD3/CD28 antibody, which is similar to T cell activation *in vivo*, IFN-γ production by CD4^+^ and CD8^+^ T cells were significantly compromised by loss of CD28 expression (**Figures 4B, C**). Similarly, degranulation capacity of CD28^-^ T cells was remarkably lower than their CD28^+^ counterparts. To exclude the impact of anti-CD28 antibody stimulation on CD28 expression, ex vivo stimulation with PMA/ionomycin was performed. As a result, loss of CD28 still led to defects in IFN-γ production and degranulation ability in both CD4^+^ and CD8^+^ T cells (**Figures 4D, E**). Further analyses were also performed to determine function of CD28^-^PD-1^+^TIGIT^+^ T cells (**Figures 4F, G**); Consistent with the total compartment, CD28^-^PD-1^+^TIGIT^+^ subset displayed a more severely dysfunctional status compared with CD28^+^PD-1^+^TIGIT^+^ subset of both CD4+ (**Figure 4H**) and CD8+T cells (**Figure 4I**). Additionally, CD28^-^PD-1^+^ and CD28^-^ TIGIT^+^ T cells also showed compromised capacity in degranulation but not IFN-γ production (**Supplementary Figures S1A–D**). These data demonstrated that loss of CD28 was associated with function impairment and could even define a severely exhausted T-cell subset.

### The improvement of T cell senescence and exhaustion is associated with disease remission after therapy

**Figure 5:**
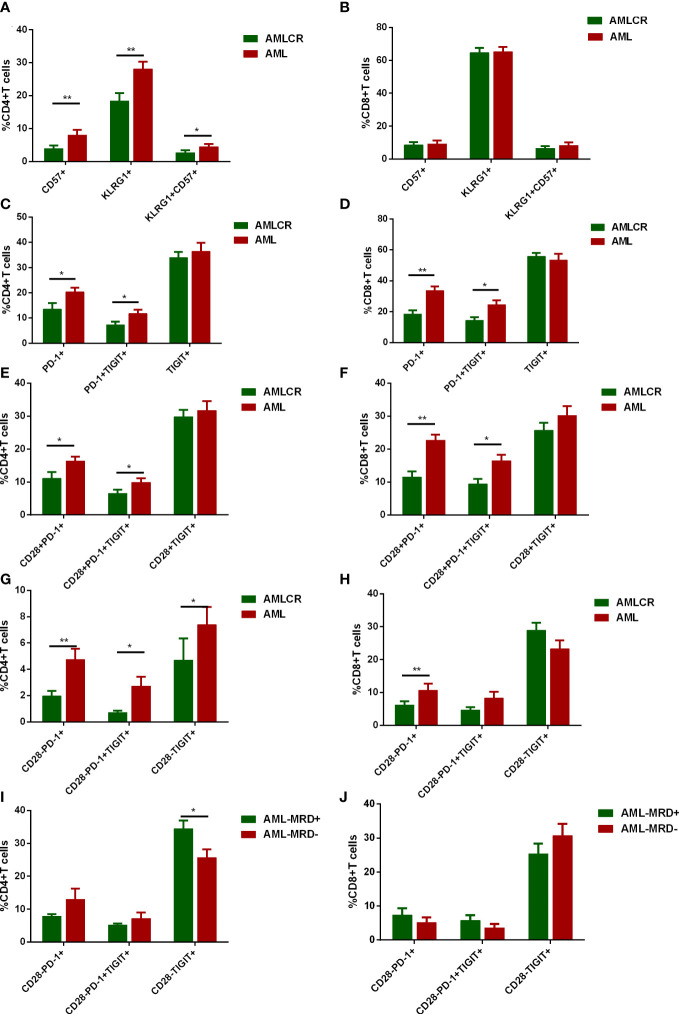
Dynamic alteration of senescent T cells and T cell exhaustion in different disease status of AML. CD57 and KLRG-1 expression of CD4^+^ T cells **(A)** and CD8^+^ T cells **(B)** were evaluated in *de novo* AML patients (n=42) and AML patients in complete remission (AML-CR, n=32). In addition, the percentages of PD-1^+^, TIGIT^+^ and PD-1^+^TIGIT^+^ subsets of CD4^+^ **(C)** and CD8^+^ **(D)** T cells in BM of *de novo* AML patients and AML-CR were compared. Further analyses of PD-1 and TIGIT expression on CD28^+^CD4^+^ **(E)** and CD28^+^CD8^+^ **(F)** T cells in BM of *de novo* AML patients and AML-CR were performed. Similarly, PD-1 and TIGIT expression on CD28^-^CD4^+^ **(G)** and CD28^+_^CD8^+^ **(H)** T cells in BM of *de novo* AML patients and AML-CR were compared. At last, the expression of PD-1 and TIGIT in CD28^-^ subsets of CD4^+^ **(I)** and CD8^+^ **(J)** T cells were analyzed in AML-CR patients with minimal residual disease (MRD) (n=12) and without MRD (n=20). Student’s t test or Mann-Whitney U test was used to analyze the data according to normal distribution of data. *, ** indicated p-values between 0.01 to 0.05, 0.001 to 0.01 respectively.

Although senescent and exhausted T cells were compared between *de novo* AML patients and HDs, whether status of T cells was improved after disease remission remains largely unclear. Senescence and exhaustion, especially the severe exhaustion of T cells was evaluated in the BM of *de novo* AML and AML patients achieving CR. The frequencies of CD57^+^, KLRG-1^+^ and CD57^+^KLRG-1^+^ subsets in CD4^+^ T cells were significantly decreased after disease remission (**Figure 5A**). Similar to HDs, AML-CR group did not show significant alteration in CD8^+^ T cells expressing either CD57 or KLRG-1 or coexpressing CD57 and KLRG-1(**Figure 5B**). As for exhausted markers, both PD-1^+^ and PD-1^+^TIGIT^+^ subsets of CD4^+^ and CD8^+^ T cells experienced a dramatically decrease after complete remission (**Figures 5C, D**). Notably, TIGIT expression was not significantly downregulated in CD4^+^ or CD8^+^ T cells (**Figures 5C, D**). Further analysis showed both CD28^-^ and CD28^+^ subpopulation of PD-1^+^ and PD-1^+^ TIGIT^+^ CD4^+^ T cells were significantly reduced after disease remission (**Figures 5E, G**). Moreover, CD28^-^ TIGIT^+^ rather than CD28^+^ TIGIT^+^ CD4^+^ T cells were decreased after remission (**Figures 5E, G**). Additionally, significant decrease of PD-1^+^TIGIT^+^CD8^+^ T cells was observed in CD28^+^ rather than CD28^-^ T cells (**Figures 5F, H**). Both CD28^-^PD-1^+^ and CD28^+^PD-1^+^ subsets of CD8^+^ T cells were significantly diminished after CR (**Figures 5F, H**). In addition, 12 patients in AML-CR group still have MRD after disease remission. Only CD28^-^TIGIT^+^CD4^+^ T cells significantly increased in these patients (**Figures 5I, J**). These data suggested that CD4^+^ T cells achieved a more comprehensive recover from senescent and exhausted status than CD8^+^ T cells after disease remission and could even be associated with MRD status.

### CD28^-^ Exhausted T cells link to lymphocyte count in peripheral blood and predict poor prognosis in *de novo* AML patients

**Figure 6:**
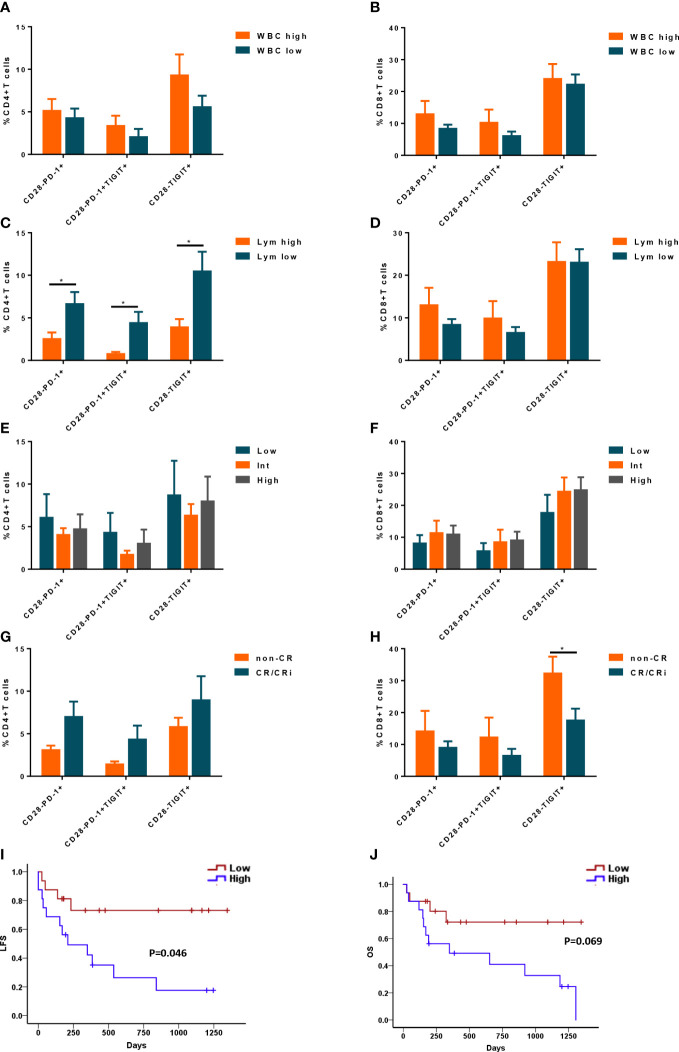
The correlation between CD28^-^ exhausted subsets and clinical features and outcome of AML patients. The percentages of CD28^-^PD-1^+^, CD28^-^PD-1^+^TIGIT^+^, and CD28^-^TIGIT^+^ subsets in CD4^+^ T cells **(A)** and CD8^+^ T cells **(B)** in BM were analyzed in AML patients with low and high level of white blood cell count (WBC) (n=22 and 20 respectively). Likewise, the percentages of CD28^-^PD-1^+^, CD28^-^PD-1^+^TIGIT^+^, and CD28^-^TIGIT^+^ subsets in CD4^+^ T cells **(C)** and CD8^+^ T cells **(D)** in BM were analyzed in AML patients with low(n=22) and high level of lymphocyte count (n= 20 respectively). Moreover, the percentages of CD28^-^PD-1^+^, CD28^-^PD-1^+^TIGIT^+^, and CD28^-^TIGIT^+^ subsets in CD4^+^ T cells **(E)** and CD8^+^ T cells **(F)** in BM were analyzed in AML patients with low (n=9), intermediate(n=20) and high (n=13) cytogenetic risk. Additionally, the correlation between CD28^-^PD-1^+^, CD28^-^PD-1^+^TIGIT^+^, and CD28^-^TIGIT^+^ subsets in CD4^+^ T cells **(G)** and CD8^+^ T cells **(H)** in BM and treatment response were evaluated (n=32). Moreover, the frequency of CD28^-^TIGIT^+^CD8^+^ T cells in BM could also predict the leukemia free survival (LFS, **I**) and overall survival (OS, **J**). Student’s t test or Mann-Whitney U test was used to analyze the frequencies of CD28- exhausted subsets in different subgroups according to normal distribution of data. Log-rank test were used to analyze the LFS and OS. * indicated p-values between 0.01 to 0.05.

Due to the heterogeneity of AML, it is critical to evaluate whether the frequencies of CD28^-^ exhausted T cells correlate with clinical and genetic features. There were no significant differences in the proportion of CD28^-^ exhausted subsets in CD4^+^ or CD8^+^ T cells between higher WBC (≥10×109/L) and lower WBC (<10×109/L) groups (**Figures 6A, B**). However, higher frequencies of CD28^-^PD-1^+^, CD28^-^PD-1^+^TIGIT^+^ and CD28^-^ TIGIT^+^ subsets of CD4^+^ T cells but not CD8^+^ T cells in BM of in *de novo* AML significantly correlated to lower lymphocyte count in peripheral blood (**Figures 6C, D**). Additionally, no difference of CD28^-^ exhausted T cells subsets was observed in low, intermediate and high cytogenetic risk group (**Figures 6E, F**). Intriguingly, only higher frequency of CD28^-^TIGIT^+^CD8^+^ T cells was observed in patients with non-CR after induction (**Figures 6G, H**). Moreover, higher frequency of CD28^-^TIGIT^+^CD8^+^ T cells at diagnosis predicted poorer outcome of AML (**Figures 6I, J**). However, CD28^-^PD-1^+^, CD28^-^PD-1^+^TIGIT^+^CD8^+^T cells did not show significant correlation with the outcome (**Supplementary Figures S2A, B**); In addition, CD28^+^PD-1^+^, CD28^+^PD-1^+^TIGIT^+^ and CD28^+^TIGIT^+^ subsets of CD8^+^ T cells also showed no significance with the outcome (**Supplementary Figures S2C–E**). These data indicated that CD28^-^TIGIT^+^CD8^+^ T cells might be a novel predictor independent of cytogenetic features for clinical response and prognosis in AML patients.

## Discussion

Although accumulation of exhausted T cells in AML was widely reported, extremely poor efficacy of anti-PD-1 therapy in AML warranted deeper phenotypical and functional analysis of T cells status. We defined severely exhausted subsets of T cells that correlated to AML occurrence and predicted the prognosis of AML by combination of senescent marker CD28 and exhausted markers like PD-1 and TIGIT. Our data provided a novel insight into T-cell exhaustion in AML patients.

Consistent with previous studies, more CD4^+^ and CD8^+^ T cells showed a shift to terminal differentiation in *de novo* AML. However, the terminal differentiation was characterized by lack of CD28 expression and decrease of Tnaive cells rather than TEM in CD8^+^ T cells, as reported by F. Brauneck (5). Different sources of T cells like BM or PB might contribute to the discrepancy. Tnaive cells are more dependent on the costimulation signaling mediated by CD28 than antigen-experienced T cells such as memory and effector T cells (15). Loss of CD28 expression is not only correlated to terminal differentiation especially in TEMRA stage but also linked to T cell senescence. Accordingly, CD28^-^ T cells accounted for a predominant part of T cells with senescent phenotype KLRG-1^+^ CD57^+^. Recently, Leo Luznik et al. demonstrated that AML blasts could induce KLRG-1 and CD57 expression on CD8^+^ T cells ex-vivo and was associated with unresponsiveness to PD-1 blockade in AML (21). However, the comparison of KLRG-1^+^CD57^+^CD8^+^ T cells in BM of AML patients and HDs was not performed in the study. In contrast, our study showed that CD4^+^ T cells rather than CD8^+^ T cells displayed higher expression of CD57 and coexpression of CD57 and KLRG-1 in BM of *de novo* AML patients. Senescence of T cells can be triggered by replicative senescence generally incurred by natural age-related process, and premature senescence induced by outside factors such as cell stress and interaction with regulatory T cells (6, 12). Considering that the eventual frequency of senescent T cells results from the multiple processes in bone marrow niches of AML, it is critical to discriminate the results of ex vivo from *in situ* of BM.

Senescence of T cells referred to an irreversible process while most exhausted T cells could be reinvigorated by ICIs (6, 12, 14). Severity of T cell exhaustion could also contribute to resistance to anti^-^PD-1 therapy (9). Severely exhausted T cells could be defined by abundance of PD-1 expression and simultaneous expression of other inhibitory receptors (9). However, due to consecutive expression of PD-1 on T cells, expression abundance evaluation is rather subjective. PD-1 and TIGIT were used to define exhausted phenotype of T cell in AML (22), However, there was no significant difference in the frequencies of CD8^+^TIGIT^+^T cells between *de novo* AML group and HDs in our study, which is not consistent with the previous report by others (22). T cells in our study were obtained from BM in AML patients and HDs, while T cells in reported study were derived from PB in HDs and BM in AML patients. Different sample sources might lead to the inconsistence.

CCR7 and CD45RA are generally used to define differentiated status of T cells. However, combination of CCR7 and CD45RA and inhibitory receptors like PD-1 and TIGIT could not well reflect the T-cell subsets correlated to AML occurrence. Kamphorst AO et al. demonstrated that rescue of exhausted T cells by anti-PD-1 therapy was CD28 dependent in mice model of life-long chronic lymphocytic choriomeningitis virus (LCMV) infection (15). However, whether CD28 expression could help to define a severely exhausted subsets remains unclear. We found that lack of CD28 expression in exhausted T cells could not only lead to phenotypical shift to the terminal stage but also result in significant functional impairments compared with CD28^+^ exhausted T cells. We also observed a senescent phenotype with higher expression of CD57 and lower expression of IL-7 receptor alpha (CD127) in CD28^-^PD-1^+^TIGIT^+^ T cells, which might restrict the capacity of self-renewal capacity and the long-term living. Notably, we found higher expression of PD-1 and TIGIT mainly distributed in CD28^-^ subsets especially in CD8^+^ compartment in *de novo* AML group. However, improvement of exhaustion status after CR mainly occurred in CD28^+^PD-1^+^TIGIT^+^ rather than CD28^-^PD-1^+^TIGIT^+^ subset of CD8^+^ T cells, neither was TIGIT^+^ T cells significantly changed after CR. Taken together, these data might provide a rational explanation for poor efficacy of PD-1 blockade in AML patients.

WBC and cytogenetic alterations were considered as the main factors impact clinical outcome (23). However, CD28^-^ exhausted subsets did not correlated to WBC or cytogenetic risk. Additionally, higher frequency of CD28^-^ exhausted subsets in CD4^+^ T cells of BM was linked to lower lymphocyte count in PB. Previous studies showed that exhaustion linked to dysfunction and apoptosis of CD4^+^ T cells in BM (24), however the mechanism of peripheral reduction of lymphocytes needs to be explored in the future. It seems that CD28^-^ exhausted subsets in CD4^+^ T cells could be more sensitive to reflect AML load than CD8^+^ T cells. Both CD28^-^PD-1^+^ and CD28^-^PD-1^+^TIGIT^+^CD4^+^ T cells were significantly decreased after CR while only CD28^-^PD-1^+^CD8^+^ T cells showed the same trend. Moreover, CD28^-^TIGIT^+^CD4^+^ T cells could even significantly elevate in AML with positive MRD. However, CD28^-^TIGIT^+^CD8^+^ T cells displayed the superior capacity to predict response to induction therapy and prognosis. Higher frequency of CD28^-^TIGIT^+^CD8^+^ T cells at diagnosis correlated to non-CR after induction and shorter LFS.

There are some limitations in our study. Despite a larger *de novo* AML cohort than previous studies, a certain proportion of patients dropped out in our study, even if this did not affect the result at diagnosis. However, the small sample size and treatment variations might lead to the biased result in survival analysis.

In summary, lack of CD28 expression could define severely exhausted subsets of T cells with terminally differentiated and senescent features. Higher level of CD28^-^ exhausted T cells correlated to AML occurrence and poor prognosis. Our findings provide a novel insight into exhausted T cells in AML and the potential combinatory strategies to improve the efficacy of PD-1 blockade.

## Data availability statement

The raw data supporting the conclusions of this article will be made available by the authors, without undue reservation.

## Ethics statement

The studies involving human participants were reviewed and approved by Institutional Human Ethics Review Committee of the First Affiliated Hospital of Xiamen University. Written informed consent to participate in this study was provided by the participants’ legal guardian/next of kin.

## Author contributions

YTH, HZ, YZ, YH, and LL performed the experiments and data analysis. ZFL, ZJL, JZ, and ZF collected bone marrow samples and clinical data. XY, LL, and BX aided in interpreting the results and worked on the manuscript. XY, LL, and BX conceived and planned the experiments. All authors contributed to the article and approved the submitted version.
