# Supplementary material for: Loss of CD28 expression associates with severe T-cell exhaustion in acute myeloid leukemia
Source: Front Immunol. 2023 Mar 7;14:1139517. doi: 10.3389/fimmu.2023.1139517 (PMC10027902; doi:10.3389/fimmu.2023.1139517)
Supplement: Supplementary file 1 [file DataSheet_1.docx]

Supplementary figure S1

Supplementary figure S1: Function analysis of CD28^-^ exhausted subsets and the counterparts of T cells in in the bone marrow (BM) of de novo AML patients. (A-B):CD107a expression and IFN-γ production were simultaneously evaluated in CD28^-^PD-1^+^ and CD28^+^PD-1^+^ subsets of CD4^+^T cells(A) and CD8^+^ T cells (B) stimulated by anti^-^CD3/CD28 antibody ex vivo. (C-D) : similar function analysis of CD28^-^TIGIT^+^ and CD28^+^TIGIT^+^ subsets of CD4^+^T cells(C) and CD8^+^ T cells (D) was also performed (n=17). *, ** and *** indicates p-values between 0.01 to 0.05, 0.001 to 0.01 and 0.0001 to 0.001, respectively.

**
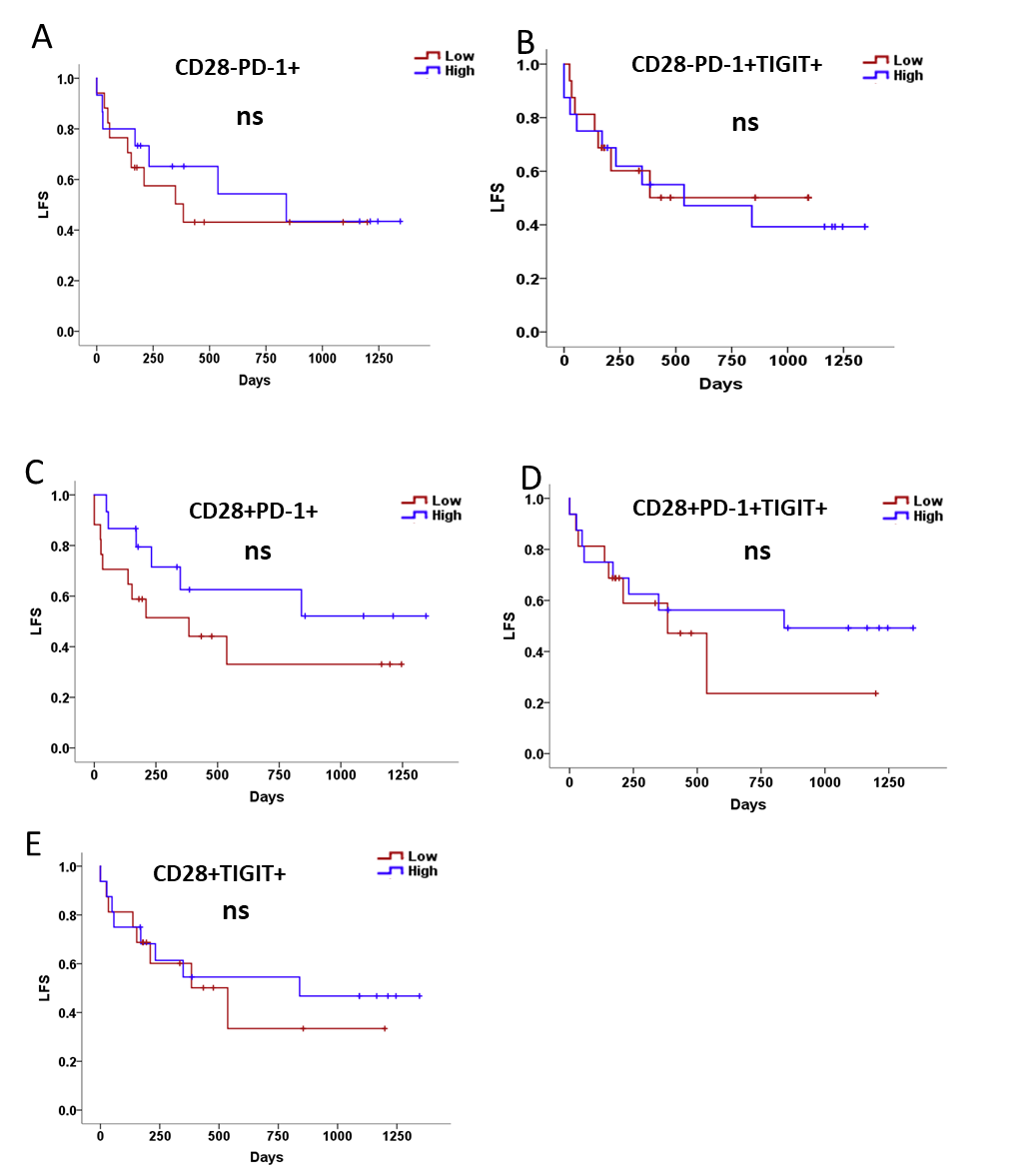
**

**Supplementary figure S2**

Supplementary figure S2: The role of exhausted subsets of CD8^+^ T cells in BM in predicting the leukemia free survival (LFS) in AML patients. The cut-off value of each subset was the median of their frequency. (A-B) the role of CD28^-^PD-1^+^(A),CD28^-^PD-1^+^TIGIT^+^ (B) subset of CD8^+^T cells in predicting LFS. (C-E) the role of CD28^+^PD-1^+^ (C), CD28^+^PD-1^+^TIGIT^+^ (D)and CD28^+^TIGIT^+^ (E) subset of CD8^+^T cells in predicting LFS. ns refers to no significant difference (p＞0.05).
